# Predicting Surgical Service Demands: A Modeling challenge using Administrative Data.

**DOI:** 10.23889/ijpds.v7i3.1931

**Published:** 2022-08-25

**Authors:** Alan Katz, Carole Taylor, Ekuma Okechukwu, Ruth-Ann Soodeen

**Affiliations:** 1 University of Manitoba; 2 MCHP

## Objectives

Publicly funded healthcare delivery systems use projections to ensure the availability of adequate future service delivery. Planning cycles need to consider infrastructure, human resources, and other essential requirements with an adequate lead time. Projections are fraught with challenges due to multiple unknowns but new developments in modeling may be useful.

## Approach

We explored the available data to determine the best approach to modeling surgical demand. The Manitoba Population Research Data Repository includes 90+ databases linkable at the person level over time. These include the population registry which includes all Manitobans registered for the universal healthcare benefit. Hospital discharge abstracts include over 20 relevant diagnoses (ICD10) and procedure codes for each admission. Medical services claims include all physician services provides with ICD 9CM codes. Fee-for-service physicians are paid based on these and alternate funded physicians are required to submit shadow claims.

## Results

We found 349,171 orthopedic procedures of which 18.1% were absent from the Medical claims files and 551,508 medical claims of which 27.5% lacked a corresponding hospital abstract. We also identified 230,717 ophthalmologic procedures in the hospital data of which 2.5% had no corresponding medical claim; of the 648,826 medical claims 66.2% had no matching hospital abstract. Resource requirements of procedures are reflected in the number and complexity of each procedure performed. Historical changes over time reflect changing demand (population growth and aging) balanced by available resources. Available resources cannot be predicted via modelling. The best fit based on the validation dataset was a Seasonal Autoregressive Integrated Moving Average model with a Mean Absolute Percentage Error (MAPE) of 5.327%. which translates to 94.7% accuracy.

## Conclusion

Despite the limitations of modeling based on past behavior, we were able to predict surgical demand with 95% accuracy. These projections are valid partly due to the persistence of historical constraints through the validation period. Policies that address these service provision limitations would precipitate a need to adjust the model.

